# Rapid strengthening of westerlies accompanied intensification of Northern Hemisphere glaciation

**DOI:** 10.1038/s41467-023-39557-4

**Published:** 2023-07-03

**Authors:** Joshua D. Bridges, John A. Tarduno, Rory D. Cottrell, Timothy D. Herbert

**Affiliations:** 1 Department of Earth and Environmental Sciences, University of Rochester, Rochester, NY 14627 USA; 2 Department of Physics and Astronomy, University of Rochester, Rochester, NY 14627 USA; 3 Laboratory of Laser Energetics, University of Rochester, Rochester, NY 14623 USA; 4 Department of Earth, Environmental and Planetary Sciences, Brown University, Providence, RI 02912 USA

**Keywords:** Palaeoclimate, Palaeoceanography, Atmospheric dynamics

## Abstract

The trigger, pace, and nature of the intensification of Northern Hemisphere Glaciation (iNHG) are uncertain, but can be probed by study of ODP Site 1208 North Pacific marine sediments. Herein, we present magnetic proxy data that indicate a 4-fold increase of dust between ~ 2.73 and ~ 2.72 Ma, with subsequent increases at the start of glacials thereafter, indicating a strengthening of the mid-latitude westerlies. Moreover, a permanent shift in dust composition after 2.72 Ma is observed, consistent with drier conditions in the source region and/or the incorporation of material which could not have been transported via the weaker Pliocene winds. The sudden increase in our dust proxy data, a coeval rapid rise in dust recorded by proxy dust data in the North Atlantic (Site U1313), and the Site 1208 shift in dust composition, suggest that the iNHG represents a permanent crossing of a climate threshold toward global cooling and ice sheet growth, ultimately driven by lower atmospheric CO_2_.

## Abstract (web summary)

The amount and composition of North Pacific dust tracked by rock magnetism suggests that the intensification of North Hemisphere Glaciation ca. 2.7 million years ago marked the permanent crossing of a climate threshold.

## Introduction

The Pliocene Epoch (5.3–2.6 Ma) is considered an analog for future climate given current anthropogenic warming because temperatures were 2–4 °C higher^1^ and atmospheric CO_2_ concentrations were similar to the present (~400 ppm)^2^. This relatively warm and humid Pliocene world deteriorated into a drier, cooler climate, with Northern Hemisphere glaciation commencing at ~3.6 Ma as inferred from *δ*^18^O data^3^. However, attention has focused on intensification of Northern Hemisphere glaciation, hereafter the iNHG, at ~2.73 Ma, recorded by decreases in biogenic opal and alkenone mass accumulation rate (MAR)^4,5^. The iNHG trigger remains unresolved. Proposals include orographic changes^6^, closure of the Panama gateway^7^, changes in orbital forcing^7^, and a reduction in atmospheric CO_2_^8^. The processes associated with these events are not mutually exclusive, but there is also a lack of agreement on whether the iNHG was gradual^3^ or abrupt^9^. While the cause of the iNHG is still debated, the event itself is well-documented. It is marked by pronounced increases in IRD at ~2.75 to 2.7 Ma in the North Pacific^10^, and in the North Atlantic^11^ and Nordic Seas^12^, with more than an order of magnitude increase recorded after ~2.72 Ma in the latter. Distinct climatological and palaeoceanographic changes are also associated with the iNHG^4,5,13–15^.

**Fig. 1:**
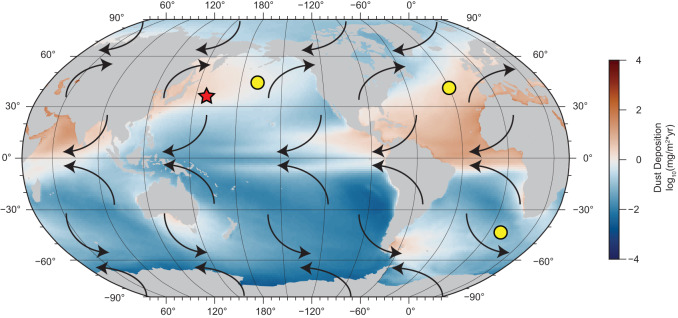
Modern dust distribution and reference sites. Red star denotes study site Ocean Drilling Program (ODP) Site 1208 and yellow circles represent marine sites with proxy dust records considered here (see text). Black arrows represent atmospheric convection cells and flow directions. Dust deposition values based on model output of C4fn simulation^81^. Base map created using PyGMT^82^.

Because of its potential to probe the climate system, dust deposition has been a focus of study^16–18^. Dust deposition can be controlled by, or itself modify changes in climate. The former reflects shifts in atmospheric circulation. The latter means dust also has a direct influence, through scattering and absorption of solar radiation^19^, or indirect influence by serving as condensation nuclei^20^. It can also strengthen the biological pump by supplying limiting nutrients like iron which can directly influence atmospheric CO_2_^21^. Here we apply environmental magnetism to trace dust deposition across the iNHG at ODP Leg 198 Site 1208 of the North Pacific (Fig. 1). Site 1208 (36.1^∘^N, 158.2^∘^E), with a water depth of 3346 m, is located on the Central High of Shatsky Rise. The site lies within the western, north-flowing side of the North Pacific subtropical gyre, known as the Kuroshio Current Extension, and is sufficiently far from the Asian continent to preclude hemipelagic inputs. The topographic height and high sedimentation rate^22^ make this site well-suited to record eolian inputs and paleoclimate. Barium records for Site 1208^16^ suggest that sulfate (SO${}_{4}^{2-}$) reduction was minimal. Therefore, magnetic records can primarily reflect external inputs rather than diagenesis^23^. Geochemical data confirm that a primary external input to the site is eolian dust from central Asia, principally from the Taklimakan and/or Gobi deserts^24,25^, with some contribution from volcanic ash.

## Results

Environmental magnetism is particularly useful in studies of dust deposition because of its potential to record changes in grain size, composition, and total flux. We must first define parameters that reflect these factors (Table 1). Below, we discuss data we have collected from Site 1208 samples (Methods) in the framework of three-time windows: 4.25–2.73 Ma (pre-iNHG), 2.73–2.68 Ma (iNHG), and 2.68–2.45 Ma (post-iNHG).

**Table 1:** Magnetic parameters for ODP site 1208

Parameter	Interpretation
Ferrimagnetic minerals	Examples include magnetite and maghemite. Structure is an inverse spinel. Magnetization arises due to antiparallel coupling of the two unequal sublattices.
Antiferromagnetic	Examples include hematite and goethite. Structure is an inverse spinel. Magnetization arises due to slight departure from antiparallel coupling of the two equal sublattices.
Mass-normalized magnetic susceptibility (*χ*)	Rough estimate of the concentration of ferrimagnetic minerals in a sample. Particularly sensitive to grains smaller and larger than 0.03 μm and 10 μm, respectively. Antiferromagnetic, paramagnetic, and diamagnetic material only dominant when ferrimagnetic material is low.
Anhysteretic susceptibility (*χ*_*ARM*_)	Dominantly reflects the concentration of small (<10 μm) magnetite grains, being particularly sensitive to grains (<0.1 μm).
Saturation Isothermal Remanent Magnetization (SIRM)	Primarily reflects the concentration of all remanence-carrying magnetic minerals–both ferrimagnetic and antiferromagnetic.
Hard IRM (HIRM)	Quantitative measure of the concentration of high-coercivity minerals (e.g., hematite).
S-Ratio (S_300_)	Provides relative proportion of high-coercivity minerals (e.g., hematite) to low-coercivity minerals (e.g., magnetite).
L-Ratio	Supports the use of the HIRM and S-Ratio environmental magnetic parameters. Relatively stable values allow for original interpretations while large fluctuations indicate possible changes in coercivities and thus provenance and grain size.
*χ*_*ARM*_/SIRM	Varies inversely with grain sizes (i.e., larger values = smaller grains). Preferentially responds to grains 1–10 μm in size. Can be affected by super- or paramagnetic material.
HIRM flux	Dust proxy presented in this paper. Higher values reflect increased fluxes of hard-coercivity material and therefore greater input of terrestrial material.

**Fig. 2:**
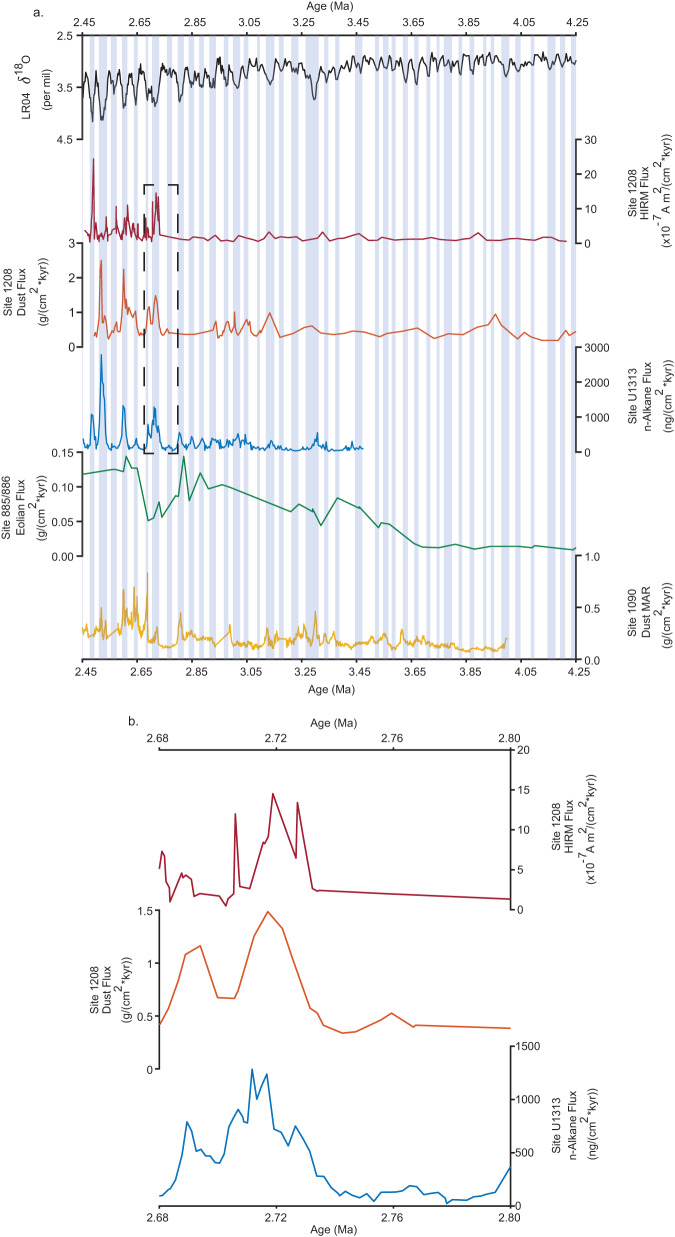
Comparison of Site 1208 dust record versus data from other sites. **a** Global benthic *δ*^18^O record^71^; Site 1208 Hard Isothermal Remanent Magnetization (HIRM) flux (this study); Site 1208 Dust flux^16^; Site U1313 n-Alkane flux^18^; Site 885/886 eolian flux^33^ and Site 1090 Fe Mass Accumulation Rate (MAR)^17^. Light blue bars represent glacial intervals. **b** Expansion (Dashed box of **a**) of records 2.68–2.8 Ma.

We start with a new dust proxy, called HIRM flux, which is particularly sensitive to the presence of hematite, a key iron oxide typically used to reconstruct eolian variations in marine cores^26^. HIRM flux is calculated by taking the product of a sample’s mass accumulation rate (MAR) and its “hard isothermal remanent magnetization" (HIRM, see Methods). An assessment of this parameter using a lower reference magnetic field (calculation of the L-ratio^27^), supports the interpretation that changes in antiferromagnetic (i.e., hematite) concentration and not grain size controls HIRM (Supplementary Figure 1; Methods). HIRM flux increases fourfold at the iNHG to a maximum of 8.8 × 10^−7^ A m^2^/(cm^2^*kyr) over ~15 kyr (Fig. 2a, b). This prominent signature reflects an abrupt deposition of primarily antiferromagnetic magnetic minerals. Increases are observed in subsequent glacials, though these are not equivalent to the iNHG event. Post-iNHG, HIRM flux values remain high, compared to their pre-iNHG counterparts.

**Fig. 3:**
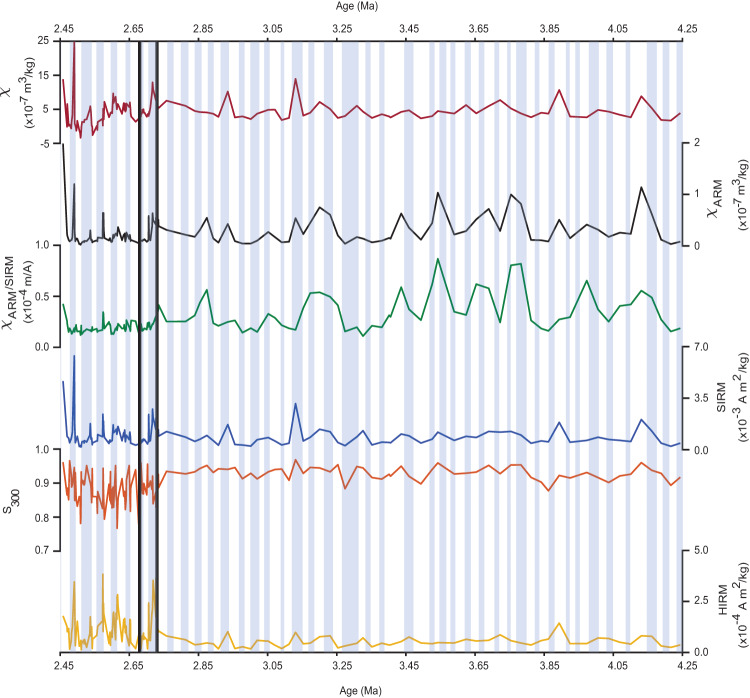
Magnetic parameters measured for Ocean Drilling Program (ODP) site 1208 sediments. Concentration (Conc), grain size (GS), and environmental (EP) proxies considered as follows: mass-normalized magnetic susceptibility (*χ*, EP); Anhysteretic magnetic susceptibility (*χ*_*ARM*_, Conc); *χ*_*ARM*_ /SIRM (Saturation Isothermal Remanent Magnetization imparted at 1.5 T, GS); SIRM (Conc); S_300_ (EP) and HIRM (Hard Isothermal Remanent Magnetization, Conc). Light blue bars represent glacial intervals. Thick black lines mark the intensification of Northern Hemisphere glaciation (iNHG) between 2.68 and 2.73 Ma.

Our magnetic proxies support this interpretation. Post-iNHG, magnetic grain size measured by *χ*_*ARM*_/SIRM (Table 1), the compositional proxy S_300_ (Table 1), and antiferromagnetic concentration (HIRM) and flux (HIRM flux, (Table 1)), differ from their pre-iNHG values (Fig. 3). Specifically, the null hypothesis that the post-iNHG values are the same as the population median can be rejected at the 95% significance level using Sign and Mood’s Median Tests. Together, these proxies reveal that after the iNHG, the magnetic population is more antiferromagnetic (e.g., hematite) dominated and also coarser grained.

**Fig. 4:**
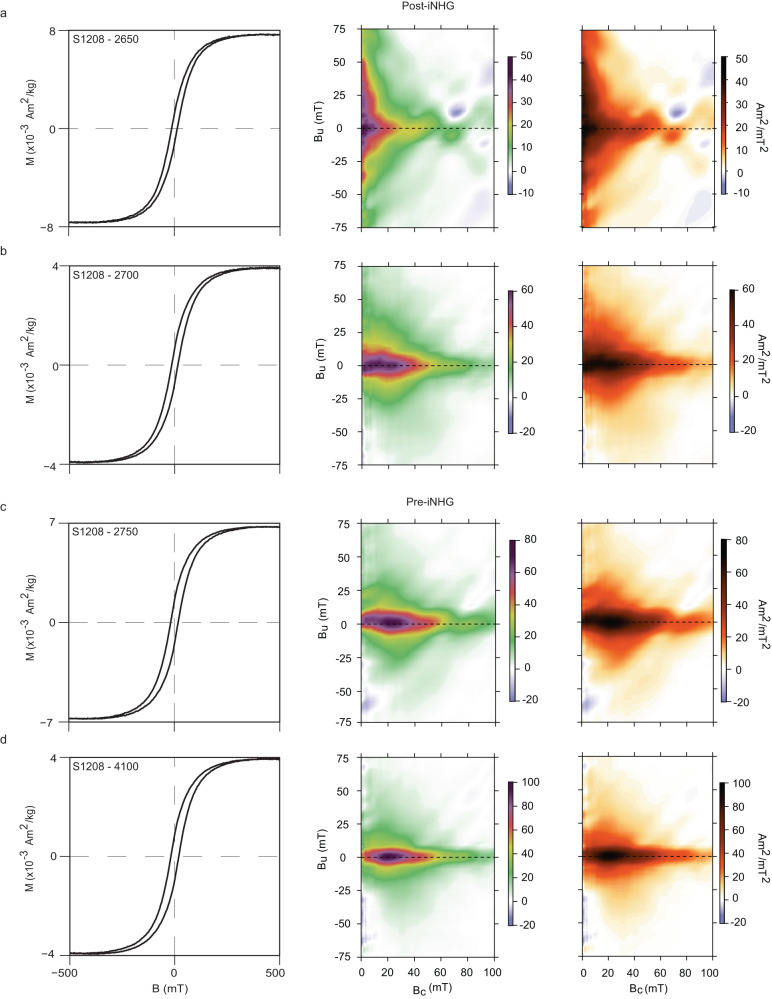
Magnetic hysteresis and First-Order Reversal Curve (FORC) analyses. Results from representative samples showing slope-corrected magnetic hysteresis curve (left) and corresponding FORC diagrams (center and right). Examples are shown for post-intensification of Northern Hemisphere glaciation (post-iNHG, **a**, **b**) and pre-iNHG (**c**, **d**) sediments.

To add further context, we measured magnetic hysteresis, first-order reversal curves (FORCs), and isothermal remanent magnetization (IRM) curves (Methods; Fig. 4; Supplementary Figure 2). Magnetic hysteresis data constrain magnetic domain state, which is related to grain size for magnetite and titanomagnetite^28,29^. Single domain (SD) grains are in general finer than pseudosingle domain (PSD) grains, which are in turn finer than multidomain (MD) grains. But for grain populations of mixed composition, as is indicated for the Site 1208 samples from our other magnetic measurements, interpretations of grain size and domain state from magnetic hysteresis data are nonunique. Nevertheless, the magnetic hystereses parameters define a nominal pseudosingle domain (PSD) state, and trend toward values consistent with larger grain sizes with time. In particular, the post-iNHG magnetic hysteresis data are shifted from the clumped pre-iNHG data (Supplementary Figure 2a). FORC analysis affords a more in-depth probe of magnetic domain characteristics, and supports the differences inferred from the initial magnetic hysteresis analysis. In particular, the pre-iNHG FORCs display fine PSD behavior^30^, whereas post-iNHG FORCs show a dominantly coarse PSD to MD signature.

Data defining the acquisition of an IRM can be decomposed into three components that provide another view of magnetic domain state Supplementary Figure 2b,c; Methods). Specifically, these data define i. low (estimated coercivity of remanence, B_1/2_ = ~11–22 mT, dispersion parameter, DP, = ~0.26–0.37), ii. middle (B_1/2_ = ~46–61 mT; DP = ~0.27–0.33 mT) and iii. high (B_1/2_ = ~141–360 mT; DP = ~0.25–0.36), magnetic coercivity components. We also note an outlier, where B_1/2_ reaches ~1 T, that may record volcanic ash. On the basis of their coercivity values, we interpret these components as i. PSD–MD magnetite, ii. SD magnetite, and iii. hematite. The IRM component importance varies across the iNHG as follows: i. 9% to 15%, ii. 77% to 75%, and iii. 15% to 10%. This analysis reveals the possibility of a nearly invariant component (ii) of relatively fine-grained magnetite. We note that similar IRM components have been found in studies of Shatsky Rise sediments younger than ~1 Ma, and the middle-coercivity component has been interpreted to reflect biogenic magnetite^31^. This might account for some of the nominally unvarying component across the iNHG in our Site 1208 IRM data. Biogenic magnetite might be less well-expressed in our other magnetic data (e.g., our FORC diagrams lack the distinct central ridge characteristic^32^ with DP < 0.2) because of abundant eolian material in an overlapping grain size range.

In summary, our magnetic data indicate that Site 1208 witnessed a pulse of hematite-rich eolian material at the iNHG, best recorded by our dust proxy (HIRM flux). This was followed by successive depositional events in glacials, consisting of a greater abundance of hematite-rich material with larger magnetic grain sizes. Concentration and flux (HIRM and HIRM flux, respectively), composition (S_300_), and grain size (*χ*_*ARM*_/SIRM) proxies support this interpretation, as do the magnetic hysteresis and FORC analyses.

## Discussion

The sudden increase in our HIRM flux dust proxy at Site 1208 indicates a profound intensification westerly winds over the North Pacific at the time of iNHG. The site lies in the westerlies, a band of winds between 30° and 60° latitude that are the primary transport mechanism by which lofted dust is deposited into the open ocean of the North Pacific^33^. Moreover, previous work in the North Pacific^16^ and North Atlantic^34^ have used equatorial shifted westerlies to explain previous dust and biological events around this time. Thus, we posit that an equatorial migration accompanied the intensification of the mid-latitude westerlies at the iNHG.

To learn more about the nature of this intensification, we compare our Site 1208 HIRM dust flux record with that of other dust proxies across the iNHG (Fig. 2a), specifically the Th-derived dust flux for Site 1208^16^, the n-Alkane flux dust for North Atlantic Site U1313 (41.00°N; 32.57°W)^18^, and the Fe mass accumulation rate reported for South Atlantic Site 1090 (42°S, 8°E)^17^. These sites provide a synoptic global view because they are at similar latitudes representing the Pacific, North Atlantic, and South Atlantic realms. We also include North Pacific Site 885/886 (44.7°N, 168.3°W)^33^, which is slightly poleward of the other sites, for comparison, (Fig. 1) but as we discuss below this site is problematic for iNHG studies.

We examine the value at the inflection point in the pre-iNHG window for each proxy, after which values continue on an increasing trend into the iNHG, with the maximum value between ~2.73 and 2.68 Ma (Fig. 2b). This interval is sufficiently broad to capture the iNHG event given differences in the resolving power of available age data at each site. We find an increase of four-times in the Site 1208 Th dust proxy, an increase of 13-times in the Site U1313 n-Alkane dust proxy, and an increase of three-times is seen in the Site 1090 Fe MAR. The changes at these sites compare well with our four-times increase in Site 1208 HIRM dust proxy. Therefore, to first-order the records at all sites point toward a large global increase of dust at these latitudes across the iNHG.

However, we also note that the increase in dust at the iNHG is less apparent (factor of only 1.4) at North Pacific ODP Site 885/886, which instead seems to record an order of magnitude increase of dust MAR starting at a nominal age of 3.6 Ma, close to the age of initial buildup of North Hemisphere ice. Environmental magnetic studies of Site 885/886 and similar sites^35–37^, however, are limited in the investigation of events like the iNHG because of low sedimentation rates and sediment types (i.e., red clay) that are problematic in terms of the high-resolution assignment. The difference in dust increase between Site 1208 and 885/886 could imply an equatorial shift^16^ of the westerlies. But we note that a recent restudy of Site 885/886^38^ has failed to confirm the original magnetostratigraphy, pointing to uncertainties in age so large that they presently preclude the use of these sediments in high-resolution paleoclimate studies. However, a shift in dust source, from the more northern Gobi Desert to the more southerly Taklimakan Desert, is consistent with our magnetic parameters and implies an equatorial migration of the westerlies around this time^16,34^(Supplementary Figure 3).

**Fig. 5:**
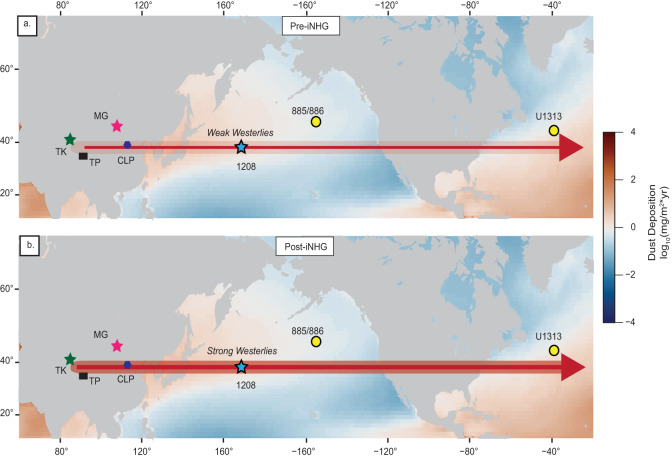
Schematic depicting the intensification of the westerlies following the intensification of Northern Hemisphere glaciation (iNHG) at ~2.73 Ma. Blue star marks study site. Yellow circles are marine sites with proxy dust records considered here. **a** Pre-iNHG climate: light shaded region in arrow represents weaker westerly wind speeds. **b** Post-iNHG climate: darkened shaded region in arrow represents intensified winds. East Asian land features: TK Taklimakan Desert (green star), MG Mongolian-Gobi Desert (pink star), TP Tibetan Plateau (black square), and CLP Chinese Loess Plateau (purple hexagon). Modern dust distribution is also shown^81^. Base map created using PyGMT^82^.

Uncertainties in the age model for ODP Site 1090^39^, and the possibility of hiatuses, also limit further comparisons with our Site 1208 record, but the age resolution at North Atlantic Site U1313 does afford the possibility of further comparisons (Fig. 2a). In particular, the Site 1208 HIRM and Th dust records, and the U1313 n-Alkane dust proxy show a coeval inflection between 2.74 and 2.73 Ma, marking the influx of dust. All three records mark a peak of dust deposition recorded by these proxies 15–30 kyr later. The rapid timescale of these changes implies the crossing of a climate threshold, signaled by stronger westerlies (Fig. 5).

The peak in dust deposition is followed by a stepped decrease recorded by the proxies thereafter. The subsequent increases in HIRM flux decreases going forward in time and occur just prior to or shortly after glacial inception. In contrast, the other records^16,18^ seem to indicate that the amplitude of dust fluxes increases forward in time, and that these dust flux events occur later in the glacial period. Hence, our records differ in timing and amplitude from those of Abell et al.^16^ and thus add new context for the iNHG.

Sources of the hematite increases observed at the iNHG and subsequent glacials are reasonably explained. The Taklimakan may have experienced an expansion and/or drying event at 2.8–2.7 Ma^40^. Additionally, deserts like the Gobi and Taklimakan are extremely arid, conditions ideal for hematite production. They are also unaffected by moisture^41^ from either the East Asian Summer Monsoon (too far inland)^42^ or Indian Monsoon (blocked by Tibetan Plateau)^43,44^. Thus, shifts in hematite formation—likely via ferrihydrite dehydration^45^—would explain this trend and the pre-iNHG muted HIRM signal.

As winds in the Pliocene are thought to have been weaker than after the iNHG^17^, they were unable to transport large quantities of hematite to Site 1208, and those magnetic grains that were transported were finer. These pre-iNHG dust events are recorded by the synchronous peaks amongst our concentration proxies. Other peaks, decoupled from HIRM, are likely the result of events not associated with atmospheric circulation shifts (e.g., biological productivity). However, given the relatively low resolution in this part of the record, we caution against overinterpretation of the data. With intensification of the westerlies at the iNHG, larger grain sizes, and thus higher concentrations of coarse-grained hematite could be carried aloft.

It is important to recognize that each of the proxies has benefits and limitations and should be expected to record slightly different aspects of the total eolian load. In particular, the benefit of magnetic measurements like HIRM flux is the ability to link to mineral type, but it might also be influenced by volcanic ash, irrespective of our efforts to avoid ash horizons during sampling. The Th-based dust flux relies on the assumption of constant influx of micro-meteoritic material, which is unknown on 10’s of kyr time scales. In addition, the n-Alkane proxy relies on the transport of plant waxes which could reflect source changes in ways different from the other proxies. As these records depend on completely independent sources, they are likely to be controlled on different time scales. There is some agreement in peak timing with those observed in the Site 1208 dust flux record^16^ and the Site U1313 n-Alkane flux^18^. However, HIRM flux dust peaks are not always coincident with these other peaks. However, these differences also highlight that any common signal seen amongst the proxies is likely a primary record of wind processes responsible for dust deposition.

Wind intensity is a function of meridional temperature gradients, with an increase resulting in stronger winds and vice-versa. In response to global cooling and ice volume increase, the high-latitudes would have cooled much greater than the tropical-subtropical regions. This would increase the meridional temperature gradient causing the winds to intensify and likely migrate equatorward.

Modeling results of the mid-Piacenzian Warm Period (MPWP, 3.3–3.0 Ma) have shown the Northern Hemisphere westerlies were located some ~4^∘^ further north of their pre-industrial locations^46^. Additionally, a weakening of the westerlies was observed in models focused on the last deglaciation^47^. Conversely, last glacial maximum (LGM) simulations in the Northern Hemisphere indicate equatorial migrations and strengthened westerlies^48^^,^^49^.

These climate changes are ultimately interwoven with changes in atmospheric CO_2_. Prior to the iNHG, CO_2_ levels remained above 300 ppm and as high as 440 ppm^2,50^^,^^51^, though there is significant scatter between these records. During this time, Site 846 *δ*^18^O records indicate at least two apparent arrested shifts toward glaciation^52^. However, modeling work suggests a 280 ppm threshold for Northern Hemisphere glaciation^53^ and it was not until marine isotope stage G6 (2.73 to 2.7 Ma) that this threshold was persistently crossed^2^. Thus, the climate threshold crossed at the iNHG implied by our observations of rapid changes of dust at the iNHG appears to be one of ice volume and atmospheric CO_2_ content in a cooling world.

It is interesting to note that historical records disagree on changes in Northern Hemisphere jet stream speeds^54–56^. Shifts in position are more robust. Historical period data indicates a poleward displacement^54,56,57^, a finding supported by the most recent IPCC report^58^. Climate simulations examining the future Northern Hemisphere jet stream behavior echo the historical findings^59–61^.

Importantly, we can reassess the interpretations of environmental magnetic records from marine and terrestrial sites in the region around Site 1208. The concentration of the 8 μm grain size fraction increases at 2.73 Ma at ODP Site 882A^62^. This increase is thought to reflect an increase in Asian aridity, despite concentration increases observed for all grain sizes present and what this means for shifts in wind strength^63^. Similarly, HIRM values from Chaona section of the Chinese loss plateau (CLP) increase markedly at 2.72 Ma^64^. This part of the record is ascribed to variations in hematite/goethite alteration, even though the increase roughly doubles over the preceding 5 Myr average. However, using the CLP for paleoclimatic studies might be problematic^65^.

Our magnetic and dust proxies are also intriguing because of the evidence they provide for a change in the nature–composition and grain size–of the eolian load at Site 1208 after the iNHG. Specifically, there is a shift toward an antiferromagnetic mineralogy (e.g., hematite) recorded by our S_300_ compositional proxy and concentration proxy HIRM, and increases in ferrimagnetic grain size (recorded by our grain size proxy *χ*_*ARM*_/SIRM). The changes recorded by these magnetic proxy data younger than 2.68 Ma (Fig. 3) are not limited to glacial periods, a finding that differs from inferences made using the Th-derived dust proxy^16^ and which is supported by our statistical analysis. Importantly, our study provides information regarding possible shifts in dust sources, something that Abell et al.^16^ does not. The magnetic data appear to record the climate system in a different way. Specifically, the shifts recorded by the magnetic proxies reflect a fundamental permanent change reflecting either a cooler, drier climate and/or the incorporation of material that could now be transported in response to the intensified westerlies.

Global cooling would reduce the amount of precipitation received by central Asia^66^ leading to increased aridification. This cooling climate will eventually lead to a switch in the contribution of ferrimagnetic minerals over antiferromagnetic minerals because the latter are preferentially formed in warmer, drier conditions^67^. The decrease in our post-iNHG HIRM flux records highlights this gradual decrease of hematite in the source regions, which will eventually be replaced with PSD and MD magnetite as the dominant magnetic contributor, as is the case today^68,69^. Also, with increased westerly winds, newly eroded ferrimagnetic material might spend less time in the source region where it might otherwise be further oxidized. Finally, we also note that the increase in ferrimagnetic grain size (recorded by our grain size proxy *χ*_*ARM*_/SIRM) indicates that winds are still intense post-iNHG, but their dust compositional load is different.

Our dust proxy, grain size, and compositional records based on environmental magnetism indicate fundamental changes at the iNHG as viewed from the North Pacific ODP Site 1208, highlighted by a prominent increase in dust deposition. This change in dust deposition is seen in sites from the North and South Atlantic, indicating its global nature. After the iNHG, the dust magnetic mineralogy at Site 1208 is more antiferromagnetic (e.g., hematite) dominated, with a coarser grain size. These changes are consistent with colder, more arid conditions and/or the incorporation of material that could not have been transported via the weaker Pliocene winds. The age sequence of dust deposition changes at the iNHG are best recorded by Site 1208 and North Atlantic Site U1313. These sites both record a distinct, coeval onset age of increased dust deposition between ~2.74 and 2.73 Ma and a peak 15–30 kyr thereafter. This temporal character indicates that the westerlies rapidly intensified at this time. Nonetheless, it is necessary to couple the intensification demonstrated here with the equatorial migration proposed elsewhere. Such an integration reveals the complete scope of paleoclimatic changes in the North Pacific around the iNHG. Combined with our statistical analysis and the greater nuances available from our high-resolution record, we posit that the iNHG represents the permanent crossing of a climate threshold defined by ice volume and decreased atmospheric CO_2_ in a cooling world. It is as yet uncertain whether a similar threshold could cause an acceleration of the weakening and poleward shift of the westerlies that is predicted by some models of future anthropogenic warming.

## Methods

### Sampling

Samples were collected from the ODP Site 1208 working half cores at the Gulf Coast core repository. Ash layers documented in the ODP Site 1208 initial reports^22^ were avoided during sampling. Samples were collected in 10 × 75 mm borosilicate glass test tubes. A Parafilm strip was placed over the test-tube end following collection to prevent drying. These tubes were subsequently trimmed at the University of Rochester so the sediment was fully exposed to the laboratory-induced fields used for our environmental magnetic proxies.

### Depth corrections

To correct for sediment expansion after recovery, we applied a modified version of the equation used by Venti and Billups^70^ to construct a Site 1208 age model spanning 1.8 to 3.7 Ma^70^. These ‘recompressed’ depths (RD) were calculated as

1 $$RD=(MPD-CTD)\cdot \left(\frac{100}{\%CR}\right)+CTD,$$

where MPD, CTD, and %CR are mid-point depth, core-top depth, and percent core recovery, respectively. MPD is the depth of the middle of the sample interval (~2 cm). CTD is the depth of the top of the core from which the sample was taken. Percent core recovery is the percentage of the 9.5 m drilling penetration that was recovered as core material. The equation (1) can also be found in our Age Model data file. This modification of the original sediment compression equation was applied to our data between ~2.45 and 3.7 Ma because the prior recompressed depths were calculated using the ‘top’ associated with the sampling interval and not the mid-point as is standard.

### Age model

We assigned ages on the basis of two previously determined age models for Site 1208. The section spanning ~2.45–3.7 Ma is based on the Site 1208 *δ*^18^O record^70^, which had been previously tuned to the global benthic *δ*^18^O stack^71^. After adjusting the reported Site 1208 depths using our ‘recompressed’ equation (1), we used linear interpolation between depth-age tie point to assign ages to our samples. Age and depth tie points from ~3.7 Ma to 4.25 Ma are from astronomically tuned paleomagnetic datums^72^.

### Magnetic measurements

All magnetic measurements were conducted in the paleomagnetic laboratories at the University of Rochester. Eleven types of magnetic data were collected in the following sequence: 1. Bulk mass-normalized magnetic susceptibility (*χ*); 2. natural remanent magnetization (NRM); 3. alternating field (AF) demagnetization of the NRM; 4. acquisition of an anhysteretic remanent magnetization (ARM); 5. demagnetization of the ARM; 6. acquisition of a saturation isothermal remanent magnetization (SIRM) in an applied field of 1.5 T; 7. acquisition of a backfield of −100 mT; 8. acquisition of a backfield of -300 mT; 9. magnetic hysteresis parameter meaurements of coercivity (H_*c*_), coercivity of remanence (H_*cr*_), saturation magnetization (M_*s*_) and saturation remanent magnetization (M_*rs*_). *10*. acquisition of isothermal remanent magnetizations (IRMs); *11*. aquisition of First-order reversal curve data (FORCs). Measurements 1–8 were collected on test-tube sediment samples.

Bulk mass-normalized magnetic susceptibility *χ* measurements were made using a KLY-4 Agico Kappabridge in a 300 A/m field. NRM measurements were performed on a 2G Enterprises (Model 755) 3-component DC SQUID magnetometer with high-resolution sensing coils in a magnetically shielded room at the University of Rochester (background field <200 nT). AF demagnetization and ARM acquisition was done using a Magnon Alternating Field Demagnetizer 300. The samples were demagnetized in 10 mT steps up to 80 mT at a decay rate of 5 mT/s. ARMs were imparted at a peak field of 80 mT and a bias field of 50 μT. SIRMs and backfields were imparted in applied fields of 1.5 T, and −100, and −300 mT, respectively, with an ASC Scientific IM-10-30 Impulse Magnetizer. Magnetic hysteresis parameters, IRM acquisition curves, and FORCs were measured on representative samples with a Princeton Measurements Corporation MicroMag 2900 Series Alternating Gradient Force Magnetometer (AGFM).

### Environmental magnetism parameters

The S-Ratio (S_300_)^73^ is defined as

2 $${S}_{300}=1-\left[\frac{IR{M}_{-300mT}}{SIRM}*\frac{1}{2}\right],$$

Hard Isothermal Remanent Magnetization (HIRM)^74^ is defined as

3 $$HIRM=\frac{(SIRM+IR{M}_{-300mT})}{2},$$

and the L-Ratio^27^ is defined as

4 $$L-Ratio=\frac{HIR{M}_{300mT}}{HIR{M}_{100mT}},$$

where HIRM_100*mT*_ is calculated similarly to HIRM_300*mT*_ but with IRM_−100*mT*_.

S_300_ and HIRM are qualitative and quantitative measures, respectively, of the antiferromagnetic contribution to the sediment. Lower S-Ratio values (<1) indicate the presence of high-coercivity material (e.g., hematite) while higher values (~1) indicate the dominance of low-coercivity material (e.g., magnetite). The L-Ratio is a check to ensure that S_300_ and HIRM can be interpreted in this way^27^. Comparison of the environmental proxies (*χ*, S_300_), concentration proxies (*χ*_*ARM*_, SIRM, and HIRM), and grain size proxy (i.e., *χ*_*ARM*_/SIRM) allow for discernment of times with greater dust deposition into the ocean.

IRM acquisition curves were decomposed using the MAX UnMix software^75^. This software requires manual ‘tuning’ of the Mean Coercivity (B_1/2_), Dispersion Parameter (DP), Relative Proportion, and Skewness to achieve a model that best represents the fitted data, in order to minimize the Residual Sum Square (RSS) value. FORCs were processed using the VARIFORC protocol^76^ within FORCinel^77^.

#### HIRM flux

HIRM flux (A m^2^/(cm^2^*kyr) was determined by multiplying HIRM (A m^2^/kg) concentration values by mass accumulation rates (MARs), which were in turn calculated by multiplying dry-bulk density (DBD, g/(cm^3^)) data^22^ by corresponding sedimentation rate values. We note that our HIRM values are mass normalized, and differentiated from other efforts that have used volume normalization^35,36^. We urge use of mass normalization following the method described above to allow future comparisons between data sets.

### Statistics

Statistical tests were applied to both the magnetic and HIRM flux dust record. Histograms were examined to determine skewness and, by extension, whether the mean or median better represented our data. Histograms were dominantly right-tailed skewed, leading to the use of the median as a better representative of central tendency. Sign and Mood’s Median test were applied to pre-iNHG, iNHG, and post-iNHG data.

The sign test^78^ is a nonparametric test that examines the null hypothesis that the values in a given population randomly fall equally above and below the median of that population (p value = 0.5). The test was implemented in MATLAB using the signtest function with the null hypothesis that the data in a particular subgroup (i.e., pre-iNHG, iNHG, post-iNHG) had a median equivalent to that of the entire parameter’s population. We implemented one-tail tests (right or left) with an alpha level = 0.1. A one-tailed test is appropriate when you are only concerned if there is a difference between populations in a particular direction and you have an assumption of what that direction might be. The choice of using a right- or left-tailed was based on whether we believed the values in the subgroup should be larger (right-tailed) or smaller (left-tailed) than the population median. The null hypothesis was rejected if the *p* value was <0.5.

Moods median test^78^ is another nonparametric test that tests the null hypothesis that the medians of two populations are equal. A critical chi^2^ value of 2.706 was used, given degrees of freedom (df) = 1 and an alpha level = 0.1. The null hypothesis was rejected if the calculated chi^2^ value was greater than the critical chi^2^ value. This test was carried out using the mediantest function [Christian Keine (2020). Moods Median Test, https://github.com/ChristianKeine/Moods-Mediantest, GitHub. Retrieved October 30, 2020] which can be downloaded from MATLAB’s file exchange webpage.

## Supplementary information

Supplementary Information

## Data Availability

All data produced for this study are available at 10.6084/m9.figshare.23147522^79^. Source data are provided with this paper.

## Source data

Source Data

## Code availability

The codes used to produce Figs. 1–3, 4a, 5 as well as Supplementary Figs. 1, 2a, and 3 in this study are available at 10.6084/m9.figshare.23147969^80^.
