# Supplementary material for: Rapid strengthening of westerlies accompanied intensification of Northern Hemisphere glaciation
Source: Nat Commun. 2023 Jul 3;14:3905. doi: 10.1038/s41467-023-39557-4 (PMC10318093; doi:10.1038/s41467-023-39557-4)
Supplement: Supplementary file 1 — Supplementary Information [file 41467_2023_39557_MOESM1_ESM.pdf]

Supplementary Information

Rapid Strengthening of Westerlies Accompanied Intensification of Northern Hemisphere Glaciation

Bridges et al.

This document contains additional figures used to inform our reconstructions of the intensification of Northern Hemisphere glaciation (iNHG) ice-sheet extents. The following figures are included:

- **Supplementary Figure 1. Determination of dominant control on HIRM.**
- **Supplementary Figure 2. Mineralogical and grain size analysis.**
- **Supplementary Figure 3. Dust source determination for pre- and post-intensification of Northern Hemisphere glaciation (iNHG) samples.**

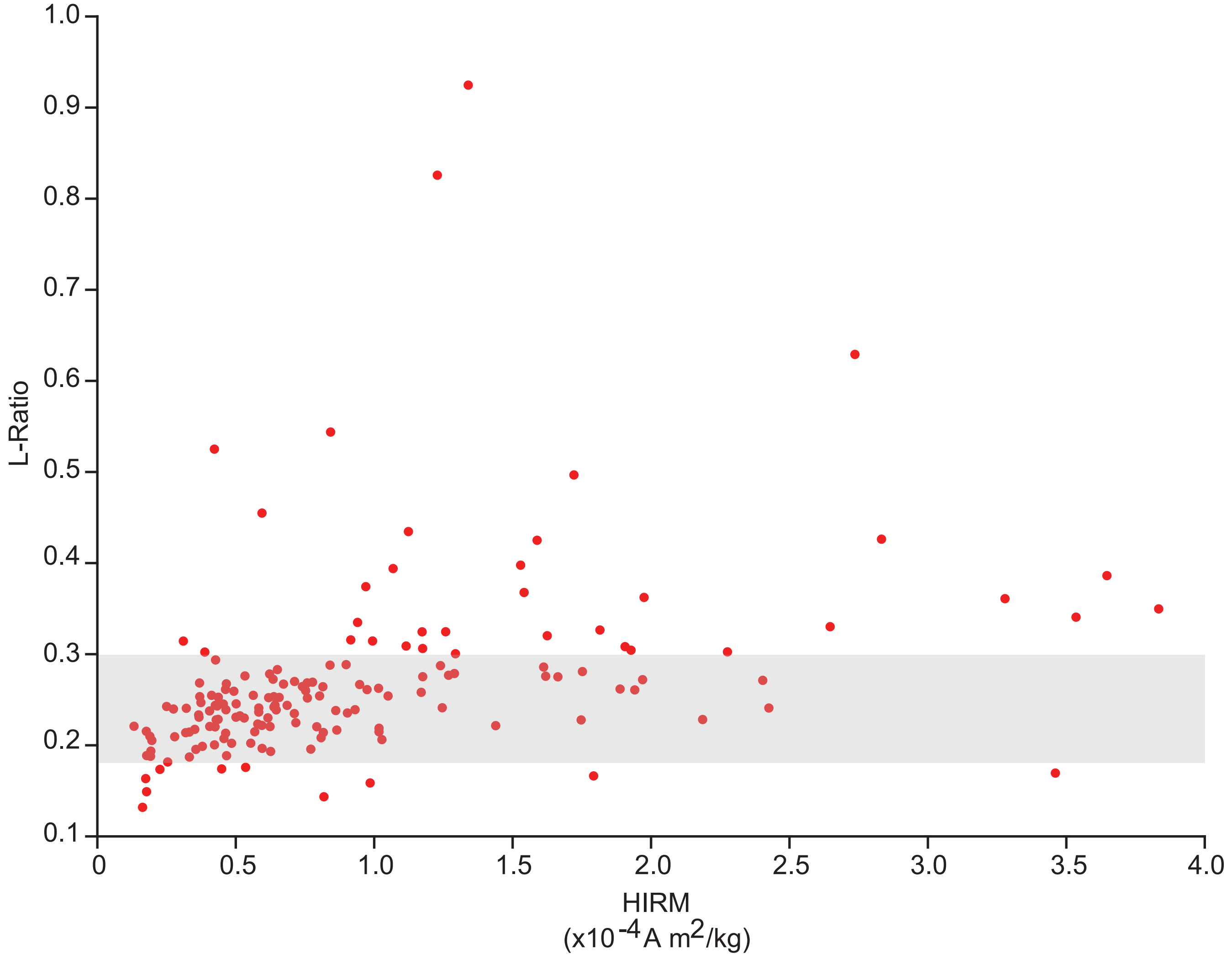

Supplementary Figure 1. Determination of dominant control on HIRM. Shaded gray box: region over which majority of samples cluster.

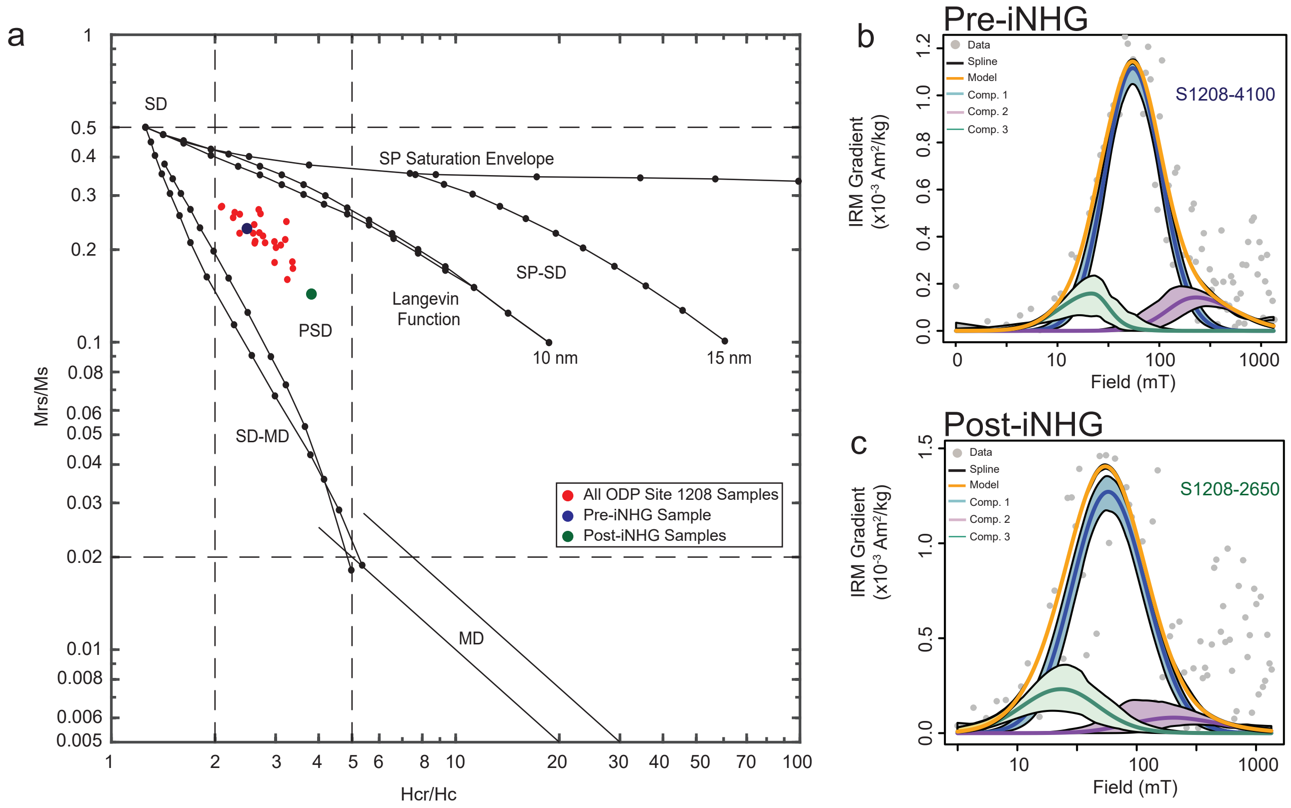

Supplementary Figure 2. Mineralogical and grain size analysis. a Day plot with calculated curves for magnetite. Domain states: SP = superparamagnetism; SD = single domain; PSD = pseudo single domain and MD = multi domain. Axis labels: Mrs = saturation remanent magnetization; Ms = saturation magnetization; Hcr = coercivity of remanence and Hc = coercivity. **b-c** Isothermal remanent magnetization (IRM) decomposition curves. Representative samples for pre-intensification of Northern Hemisphere glaciation (iNHG, b, blue circle in (a)) and post-iNHG (c, green circle in (a)).

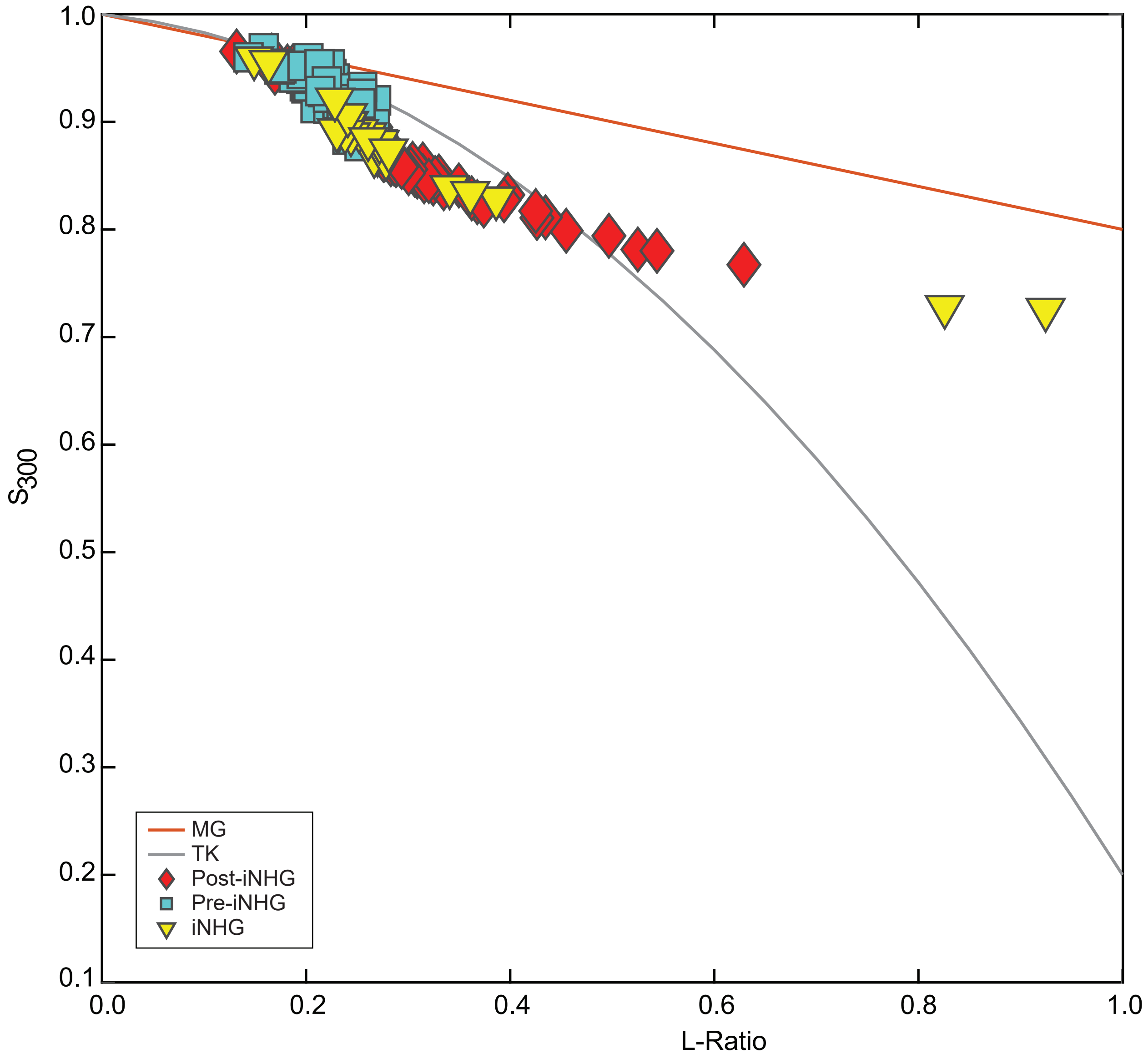

Supplementary Figure 3. Dust source determination for pre- and post-intensification of Northern Hemisphere glaciation (iNHG) samples. MG = Mongolian-Gobi Desert and TK = Taklimakan Desert. Orange and gray curves are best-fit lines for S_{300} and L-Ratio data collected on MG and TK surface samples[1], respectively. Data points spread across both MG and TK pre-iNHG but plot closer to TK best-fit line post-iNHG.

1. Q. Liu, Y. Sun, X. Qiang, R. Tada, P. Hu, Z. Duan, Z. Jiang, J. Liu, and K. Su, Characterizing magnetic mineral assemblages of surface sediments from major Asian dust sources and implications for the Chinese loess magnetism, Earth, Planets and Space 67, 1-17 (2015).
